# Supplementary material for: Real-World Evidence on Adverse Events and Healthcare Resource Utilization in Patients with Chronic Lymphocytic Leukaemia in Spain Using Natural Language Processing: The SRealCLL Study
Source: Cancers (Basel). 2024 Nov 29;16(23):4004. doi: 10.3390/cancers16234004 (PMC11639754; doi:10.3390/cancers16234004)
Supplement: Supplementary file 1 [file cancers-16-04004-s001.zip › cancers-3326009-supplementary.pdf]

Supplementary materials

Supplementary Table S1. Evaluation metrics of *EHRead®* performance identifying key variables

	Recall	Precision	F1-score
Chronic lymphocytic leukemia	0.987603	0.956	0.971545
Idelalisib	0.916667	0.982143	0.948276
Tumor lysis syndrome	0.914634	0.9375	0.925926
ECOG	0.949495	0.895238	0.921569
Ibrutinib	0.904762	0.853933	0.878613
Night sweat	0.877551	0.834951	0.855721
Bone marrow biopsy	0.795455	0.826772	0.810811
Obinutuzumab†	0.736842	0.293194	0.419476

†Reading of the term "obinutuzumab" was suboptimal, since the acronym "O" was included as a synonym of this variable, leading to many false positives during *EHRead®* detection.

Supplementary Table S2. Follow-up characteristics of CLL patients stratified by groups.

	**W&W (n=270)**	**1L Treatment (n=230)**	**2L Treatment (n=58)**
Time (months) of follow-up			
Mean (SD)	14.4 (9.6)	10.8 (8.4)	9.6 (8.4)
Median (Q1,Q3)	14.4 (6.0, 22.8)	8.4 (2.4, 15.6)	6 (2.4, 12.0)
Cause of end of follow-up			
Loss of FUP* n (%)	42 (15.6)	66 (28.7)	17 (29.3)
Death** n (%)	8 (3.0)	37 (16.1)	5 (8.6)

* Patients whose last report was collected more than one year before the end of the study period; ** All recorded intra-hospital death.

Supplementary Table S3. Summary of events and adverse events stratified by groups throughout the entire study period in patients with CLL.

	W&W (n=270)	1L Treatment (n=230)	2L Treatment (n=58)
Patients with any adverse event	195 (72.2)	203 (88.3)	46 (79.3)
**Cardiovascular**			
Cardiac arrhythmia	38 (14.1)	35 (15.2)	4 (6.9)
Atrial fibrillation	19 (7.0)	23 (10.0)	4 (6.9)
Atrial flutter	6 (2.2)	8 (3.5)	0 (0)
Hypertension	39 (14.4)	32 (13.9)	3 (5.2)
**Bleeding**			
Major bleeding	13 (4.8)	18 (7.8)	4 (6.9)
Non-major bleeding	18 (6.7)	24 (10.4)	6 (10.3)
**Second primary malignancies***			
Haematological malignancies			
Richter´s Syndrome	2 (0.7)	23 (10.0)	4 (6.9)
Hodgkin lymphoma	4 (1.5)	3 (1.3)	1 (1.7)
Non-Hodgkin lymphoma	4 (1.5)	8 (3.5)	4 (6.9)
B-cell lymphomas	1 (0.4)	1 (0.4)	1 (1.7)
Marginal zone lymphoma (MZL)	4 (1.5)	5 (2.2)	1 (1.7)
Follicular lymphoma	2 (0.7)	3 (1.3)	0 (0)
Splenic lymphoma with villous lymphocytes (SLVL)	1 (0.4)	2 (0.9)	1 (1.7)
Solid malignancies			
Breast	2 (0.7)	0 (0)	0 (0)
Lung	4 (1.5)	4 (1.7)	0 (0)
Bladder	1 (0.4)	0 (0)	0 (0)
Prostate	1 (0.4)	2 (0.9)	0 (0)
Mouth	1 (0.4)	0 (0)	0 (0)
Stomach	1 (0.4)	0 (0)	0 (0)
Colon	3 (1.1)	1 (0.4)	1 (1.7)
Pancreas	0 (0)	1 (0.4)	0 (0)
Biliary tract	0 (0)	1 (0.4)	0 (0)
Skin	14 (5.2)	11 (4.8)	4 (6.9)
Malignant melanoma of skin	4 (1.5)	4 (1.7)	0 (0.0)
**Immune, blood and lymphatic system**			
Anaemia	92 (34.1)	144 (62.6)	38 (65.5)
Thrombocytopenia	58 (21.5)	110 (47.8)	31 (53.4)
Neutropenia	14 (5.2)	32 (13.9)	10 (17.2)
Febrile neutropenia	5 (1.9)	14 (6.1)	6 (10.3)
**Respiratory**			
Pneumonitis	25 (9.3)	29 (12.6)	7 (12.1)
**Infectious diseases**			
Pneumonia	21 (7.8)	28 (12.2)	7 (12.1)
Urinary tract infectious disease	19 (7.0)	12 (5.2)	5 (8.6)
Cellulitis	5 (1.9)	11 (4.8)	3 (5.2)
Bacteraemia	8 (3.0)	18 (7.8)	8 (13.8)
Hepatitis B infection	8 (3.0)	14 (6.1)	0 (0.0)
Cytomegalovirus infection	11 (4.1)	25 (10.9)	5 (8.6)

Herpes zoster infection	5 (1.9)	13 (5.7)	2 (3.4)
Cholangitis	2 (0.7)	0 (0)	0 (0)
Meningitis	3 (1.1)	3 (1.3)	0 (0)
Septic shock	2 (0.7)	8 (3.5)	2 (3.4)
Pneumocystis jirovecii infection	1 (0.4)	1 (0.4)	0 (0)
Progressive multifocal leukoencephalopathy (PMLE)	0 (0)	1 (0.4)	0 (0)
**Gastrointestinal and hepatobiliary**			
Diarrhea	28 (10.4)	38 (16.5)	4 (6.9)
Vomiting	22 (8.1)	28 (12.2)	2 (3.4)
Nausea	24 (8.9)	26 (11.3)	7 (12.1)
Constipation	13 (4.8)	19 (8.3)	1 (1.7)
Hepatotoxicity	1 (0.4)	0 (0)	1 (1.7)
**Nervous system**			
Headache	21 (7.8)	12 (5.2)	3 (5.2)
Stroke	38 (14.1)	25 (10.9)	5 (8.6)
Transient ischaemic attack	7 (2.6)	10 (4.3)	2 (3.4)
**Skin and subcutaneous tissue**			
Rash	11 (4.1)	22 (9.6)	4 (6.9)
Alopecia	0 (0)	2 (0.9)	0 (0)
**Musculoskeletal and connective tissue**			
Arthralgia	15 (5.6)	14 (6.1)	2 (3.4)
Muscle spasms	1 (0.4)	5 (2.2)	1 (1.7)
Myalgia	5 (1.9)	8 (3.5)	3 (5.2)
**General**			
Tumour lysis syndrome	2 (0.7)	12 (5.2)	8 (13.8)
Toxicity**	9 (3.3)	23 (10.0)	5 (8.6)
Asthenia	70 (25.9)	69 (30.0)	13 (22.4)
Anorexia	14 (5.2)	25 (10.9)	5 (8.6)
Fever***	53 (19.6)	40 (17.4)	3 (5.2)
Allergic reaction / Anaphylaxis	2 (0.7)	6 (2.6)	1 (1.7)
Peripheral edema	2 (0.7)	11 (4.8)	1 (1.7)
Perfusion-related reactions	0 (0)	1 (0.4)	0 (0)

All results are shown as n (%). The presence of each feature was analysed from 1L treatment initiation to the last follow-up (1L). The presence of each feature was analysed from 2L treatment initiation to the last follow-up (2L). Adverse events were detected by temporally linking an event to the treatment period: differentiating between chronic, disease-related, or treatment-related adverse events might not be possible; to address this issue, we discarded events that were already present at baseline. * No cases of other second primary malignancies such as central nervous system, uterine cervix, thyroid gland, kidney, tongue, pharynx and larynx were identified. **Toxicity was detected as a term itself, without additional information.*** Fever was detected for any cause, not specifically due to infection or cancer.
